# Single-nucleus chromatin landscape dataset of mouse brain development and aging

**DOI:** 10.1038/s41597-024-03382-1

**Published:** 2024-06-12

**Authors:** Yuting Ma, Sicheng Guo, Yixi Chen, Yushan Peng, Xi Su, Hui Jiang, Xiumei Lin, Jianguo Zhang

**Affiliations:** 1 College of Life Sciences, University of Chinese Academy of Sciences, Beijing, 100049 China; 2 Hebei Industrial Technology Research Institute of Genomics in Maternal & Child Health, Clin Lab, BGI Genomics, Shijiazhuang, 050035 China; 3 BGI Genomics, Shenzhen, 518083 China; 4 BGI Research, Shenzhen, 518083 China; 5 School of Biology and Biological Engineering, South China University of Technology, Guangzhou, China; 6 School of Public Health, Hebei Medical University, Shijiazhuang, 050017 China

**Keywords:** Epigenomics, High-throughput screening, Epigenetic memory, Senescence

## Abstract

The development and aging of the brain constitute a lifelong dynamic process, marked by structural and functional changes that entail highly coordinated cellular differentiation and epigenetic regulatory mechanisms. Chromatin accessibility serves as the foundational basis for genetic activity. However, the holistic and dynamic chromatin landscape that spans various brain regions throughout development and ageing remains predominantly unexplored. In this study, we employed single-nucleus ATAC-seq to generate comprehensive chromatin accessibility maps, incorporating data from 69,178 cells obtained from four distinct brain regions – namely, the olfactory bulb (OB), cerebellum (CB), prefrontal cortex (PFC), and hippocampus (HP) – across key developmental time points at 7 P, 3 M, 12 M, and 18 M. We delineated the distribution of cell types across different age stages and brain regions, providing insight into chromatin accessible regions and key transcription factors specific to different cell types. Our data contribute to understanding the epigenetic basis of the formation of different brain regions, providing a dynamic landscape and comprehensive resource for revealing gene regulatory programs during brain development and aging.

## Background & Summary

The brain maturation and ageing processes are intricate and enduring phenomena that are intricately influenced by a combination of genetic and environmental factors. Although the inherent DNA composition of cells remains relatively stable throughout an individual’s lifespan, there is a continual modulation of epigenetic activities that intricately regulate the trajectory of cellular differentiation^1^. During the early developmental stages, progenitor cells meticulously modulate gene expression by accessing distinct cis-regulatory elements (CREs) during critical developmental windows, thereby recruiting trans-acting factors (TAFs). This orchestrated process plays a pivotal role in shaping functionally specific cell types and the intricate structures of the brain. Key developmental phases, such as embryonic development, puberty, and adolescence, represent critical periods characterised by heightened susceptibility to environmental influences, further emphasising the dynamic interplay of genetic and environmental determinants. Anomalies in epigenetic modifications during these transitional periods may precipitate severe neurodevelopmental anomalies and behavioural disorders^1^. Additionally, the cumulative occurrence of disturbances and errors in epigenetic regulation across a substantial population of cells within ageing brain tissue over time can contribute to deviations in health trajectories and the manifestation of neurodegenerative diseases^2,3^. Consequently, it is evident that epigenetic activities play a pivotal and intricate role in both the development and functional execution of the brain. A comprehensive understanding of the dynamic shifts in gene regulation during brain development and ageing is imperative to unravel the underlying mechanisms associated with neurodevelopmental disorders and age-related neurological conditions.

Chromatin accessibility serves as a fundamental component in the orchestration of transcriptional regulation and epigenetic control. Extensive research has demonstrated the presence of considerable epigenetic priming that occurs prior to the commitment to cellular lineages during the process of differentiation. This priming involves the initial opening of CREs before the recruitment of TAFs and the initiation of gene transcription^4,5^. As such, it is imperative to investigate the overarching open state of CREs in the brain across diverse developmental and ageing stages. This exploration is crucial to gain a deeper understanding of the molecular mechanisms underlying both the developmental progression and functional execution of the brain. Moreover, it provides a pivotal reference point for elucidating various neurodevelopmental disorders and neurodegenerative conditions. Regrettably, the existing literature lacks a comprehensive view of the dynamic landscape of global chromatin accessibility encompassing multiple brain regions from development to ageing.

Single-cell assay for transposase-accessible chromatin with sequencing (scATAC-seq) facilitates the genome-wide identification of chromatin accessibility patterns at the individual cell level. Its attributes, including high throughput, precision, and minimal sample input requirements, have positioned it as the predominant technique for investigating chromatin openness^6^. scATAC-seq allows for the concurrent exploration of the chromatin landscape in tens of thousands of individual cells, presenting an unparalleled opportunity to comprehensively characterise the epigenetic diversity within distinct brain cell populations and to elucidate the epigenetic regulatory mechanisms governing the development of diverse brain structures. Utilising model mice across various developmental and ageing stages addresses challenges associated with difficulties in brain tissue sampling and ensures sample freshness. This approach represents an invaluable resource and reference for tracing the entire lifespan of the brain.

**Fig. 1:**
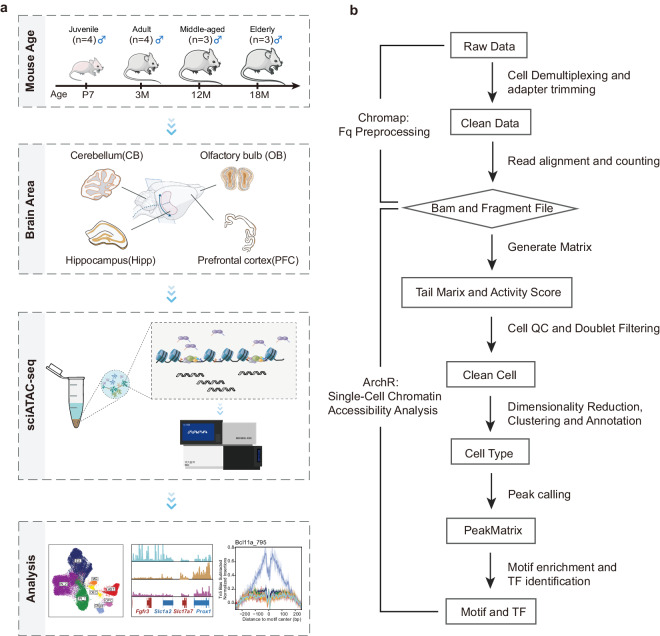
Schematic representation of experimental and data analysis procedures for single-nucleus assay for transposase-accessible chromatin with sequencing (sciATAC-seq) in the mouse brain. (**a**) The major experimental and analytical processes. Samples from mice at four developmental stages (postnatal day 7 [7 P], 3 months [3 M], 12 months [12 M], and 18 months [18 M]) were collected from four distinct brain regions (cerebellum [CB], hippocampus [HP], olfactory bulb [OB], and prefrontal cortex [PFC]) for single-cell combinatorial indexing assay for transposase-accessible chromatin with sequencing (sciATAC-seq). (**b**) sciATAC-seq workflow.

In this study, we employed the single-cell combinatorial indexing assay for transposase-accessible chromatin with sequencing (sciATAC-seq)^7,8^ to conduct genome-wide chromatin accessibility sequencing on 6,9178 cell nuclei obtained from four principal brain regions^9^ during critical developmental and ageing intervals in mice. Specifically, the selected time points for analysis included the juvenile (postnatal day 7 [7 P]), adult (3 months [3 M]), middle-aged (12 months [12 M]), and elderly (18 months [18 M]) stages (Fig. 1a). The brain regions subjected to analysis are the prefrontal cortex (PFC), governing higher cognitive functions^10^; the cerebellum (CB), implicated in motor control and balance functions^11^; the hippocampus (HP), linked to learning, memory, and spatial localisation functions^12^; and the olfactory bulb (OB), responsible for adult brain neurogenesis^13,14^. We performed chromatin accessibility profiling at the single-cell level, enabling the identification of various cell types, including excitatory neurons (EX), inhibitory neurons (IN), astrocytes (AST), microglia (MG), oligodendrocytes (oligo), oligodendrocyte progenitor cells (OPC), and olfactory ensheathing glia (OEC). Our investigation unveiled dynamic patterns of chromatin accessibility at the single-cell resolution across distinct stages of brain development and in diverse brain regions, thus furnishing a comprehensive repository for unravelling the epigenetic mechanisms governing brain development and ageing across the entire lifespan.

## Methods

### Experimental animals and samplings

The protocol was approved by the Animal Care and Use Committee at the University of Science and Technology of China (Approval ID: 2023-N (A) −513). Tissue samples were procured from distinct brain regions (OB, CB, PFC, and HP) at 7 P, 3 M, 12 M, an 18 M. After surface cleansing with phosphate-buffered saline (PBS; Meilunbio, MA0015) and elimination of residual liquid using lint-free paper, the specimens were carefully transferred to 2 mL centrifuge tubes (AXYGEN, 28820394). The samples were rapidly frozen in liquid nitrogen and stored at −80 °C for subsequent analysis.

### Nuclear isolation from brain tissue

The brain tissue was thawed at 4 °C and then cut into small fragments using surgical scissors. Subsequently, the tissue was resuspended in 2 mL of tissue lysis buffer with 320 mM sucrose, 0.1 mM EDTA, 0.1% NP40, 5 mM CaCl_2_, 3 mM MgCl_2_, 10 mM Tris (pH 7.8), 1 × complete protease inhibitors (Roche), and 167 μM β-mercaptoethanol (all dissolved in water). The suspension was transferred to a 2 mL Dounce tissue homogeniser (Kimble, No. 885300-0002). The tissue fragments were macerated on ice with the A pestle for 10–15 repetitions. Next, the resulting tissue homogenate was filtered using a 70 µm cell strainer (Corning, 352350). Following the initial filtration, the sample was macerated with the B pestle for 10–15 repetitions; the homogenate was filtered through a 40 µm cell strainer (Falcon, 352340) into a 1.5 mL tube. The tube was centrifuged at 1,200 *g* for 5 minutes at 4 °C to isolate the cell nuclei. The pellet was washed once with 1 × PBS containing 1% bovine serum albumin (BSA) and 0.2 U/μL RNase inhibitor. Finally, the pellet was resuspended in 1 × PBS containing 1% BSA in preparation for subsequent flow cytometry analyses.

### sciATAC-seq library construction and sequencing

The sciATAC-seq library was prepared as described in the published guidelines^15^. In summary, cellular nuclei were initially labelled with 3 μM DAPI and subsequently sorted into pre-prepared 96-well plates featuring distinct Tn5 tags, employing a BD FACSAria III flow cytometer. Each well accommodated 2,500–3,000 intact cell nuclei. Post-sorting, the 96-well plates were incubated at 55 °C for 30 minutes, followed by the addition of 10 μL of 40 mM EDTA to halt the transposition reaction. The sorted cell nuclei were retrieved from the plates, diluted in 1 × PBS containing 5% BSA and 5 mM EDTA, and subjected to a second DAPI staining. Subsequently, the nuclei were sorted into a second set of 96-well plates containing a polymerase chain reaction (PCR) indexing amplification reaction mix, with 25 nuclei per well. Following this sorting step, 1 μL of 0.2% Single-cell SDS lysis buffer (SDS) was introduced into each well and incubated at 55 °C for 7 minutes to lyse the cells. This was followed by the addition of 1 μL of 10% Triton-X to each well, with a subsequent incubation at room temperature for 5 minutes to terminate the SDS reaction. Ultimately, 10 μL of 2x NEBNext (NEB, M0541L) was added to each well, thoroughly mixed, and subjected to PCR amplification. Post-amplification, the PCR products underwent purification using 1 × Ampure magnetic beads, and the resulting products were processed for MGI DNBSEQ sequencing.

### Bioinformatics preprocessing

#### Processing of the original sciATAC-seq data

Chromap^16^ is an ultra-fast method utilized for aligning and preprocessing high-throughput chromatin profiles. It enables trimming sequence adapters, mapping reads to a reference genome, correcting barcodes, removing duplicates, executing Tn5 shifting, and ultimately generating fragment files. To conform to Chromap’s input standards and streamline the upstream alignment process, we converted the 40 bp cell barcodes from sciATAC to a set of 20 bp barcodes.

The cell barcodes in the raw sequencing data were defined by regions spanning 1–10 nucleotides (nt) and 32–41 nt in read 1, as well as 1–10 nt and 38–47 nt in read 2 (refer to Supplementary Table 1). The adapter sequences used for ligation were identified in sections spanning 11–31 nt in read 1 and 11–37 nt in read 2. Concurrently, segments 42–100 nt in read 1 and 48–100 nt in read 2 represented the DNA capture sequences. To establish a unique barcode combination, sequences spanning 1–10 nt and 32–41 nt from read 1, and 1–10 nt and 38–47 nt from read 2, were extracted. Subsequently, adapter sequences from both read 1 and read 2 were removed. As the barcode length reached 40 base pairs (bp), a replacement procedure ensued, substituting it with corresponding 20 bp new barcodes. These newly generated barcodes were then prepended to the beginning of the read 1. The modified FASTQ files were structured such that for read 1, the initial 20 bp represented the barcode, and the subsequent 50 bp (from 21 to 70) denoted the DNA capture sequences. In the case of read 2, the sequence consisted of the 46 bp DNA capture sequence.

Next, we performed preprocessing and mapping of reads according to Chromap’s default parameters (Minimizer k-mer length = 17, Minimizer window size = 7, and Minimum fragment length used for mapping = 30). Ultimately, we converted the original “fastq” files into a “fragment” file format readable by the R package ArchR (version 1.0.2)^17^.

#### Genome annotations and quality control of sciATAC-seq data

ArchR (version 1.0.2)^17^ necessitates gene and genome annotations to perform various tasks such as computing TSS enrichment scores, nucleotide content, and gene activity scores. The precompiled mm10 genome version in ArchR utilizes the following annotations: “BSgenome.Mmusculus.UCSC.mm10”, “TxDb.Mmusculus.UCSC.mm10.knownGene”, “org.Mm.eg.db,” and a merged blacklist obtained through the “ArchR::mergeGR()” function. This blacklist comprises regions from mm10 v2 blacklist and mitochondrial regions exhibiting high mappability to the mm10 nuclear genome, as identified. Genomic regions within the blacklist are excluded from annotation and subsequent downstream analyses.

ArchR (version 1.0.2)^17^ was employed for cell quality control, incorporating stringent criteria. Specifically, cells deemed to be of low quality were filtered out based on the following metrics: the requirement for a minimum count of unique fragments (nFrags) ≥ 1000, and a transcription start site (TSS) score per library of ≥4. Additionally, we utilized ArchR’s “addDoubletScore” function to perform individual library-level doublet removal. The principle of doublet removal involves iteratively combining different cells in the data to generate simulated doublets, which are then projected onto UMAP. Through successive iterations of this step, cells that closely resemble simulated doublet signals are identified and deemed as doublets.

The specific formula used in ArchR for doublet calculation is: Maximum number of doublets = filterRatio * total number of cells * 0.05. The original doublet rate in the sciATAC raw data was approximately 10%. Therefore, according to the formula, the parameter filterRatio should be set to 2.

#### Matrix generation of sciATAC-seq data

ArchR (version 1.0.2)^17^ was employed for cell quality control, incorporating stringent criteria. Specifically, cells deemed to be of low quality were filtered out based on the following metrics: the requirement for a minimum count of unique fragments (nFrags) ≥ 1000, and a transcription start site (TSS) score per library of ≥4. Subsequently, the ‘addDoubletScores’ function was employed to calculate doublet scores, with the filterRatio parameter set to 1.5. This parameterisation facilitated the discernment and subsequent elimination of putative doublets from the dataset.

ArchR (version 1.0.2)^17^ was used to derive TailMatrix and GeneScoreMatrix from the sciATAC-seq data. TailMatrix was constructed to assess the chromatin accessibility of individual cells within predefined genomic windows. Specifically, a 500 bp window spanning the entire genome was defined to generate a chromatin accessibility matrix based on a cell-by-fixed-length window paradigm. ArchR (version 1.0.2) utilises a weighted average of peak accessibility, with the weight assigned to each peak determined by its proximity to the TSS. The GeneScoreMatrix was established using the ‘addGeneScoreMatrix’ function to predict gene activity scores for each gene within the cells, resulting in a cell-by-gene matrix of gene activity scores. Given the inherent sparsity of sciATAC-seq data, the Markov affinity-based graph imputation of cells (MAGIC) method was implemented. This method leverages shared information among neighbouring similar cells to enhance signal strength and reduce noise, effectively imputing gene activity scores for a more comprehensive assessment.

#### Latent semantic indexing (LSI) clustering of sciATAC-seq data

LSI dimensionality reduction was executed using the ‘addIterativeLSI’ function available in the ArchR software^17^. For data clustering, the Seurat package’s ‘FindClusters’ function was employed with specific parameters: reducedDims = ‘IterativeLSI’, method = ‘Seurat’, and resolution = 0.3. The identification of marker genes for each cluster was accomplished using the ‘getMarkerFeatures’ function, considering the criteria of a false discovery rate (FDR) ≤ 0.05 and a log2 fold change (log2FC) ≥ 0.5. Cell types were annotated based on well-established cell type–specific markers. Visualisation of the clustering results in a two-dimensional space was achieved using the ‘addUMAP’ function. Lastly, Pearson correlation coefficients were calculated for the Tilematrix of different libraries to assess the similarity among libraries from the same brain region and age stage. This step aimed to ensure the authenticity and reproducibility of the sequencing data.

#### Peak calling of sciATAC-seq

Model-based ChIP-seq analysis (MACS2)^18^, accessible at https://github.com/hbctraining/Intro-to-ChIPseq, was employed to conduct peak calling for Tn5-corrected insertions, which represent each end of the Tn5 in each distinct cell type. The generation of pseudo-bulk replicates for each cell type was facilitated by using the ‘addGroupCoverages’ function. Subsequently, the identification of peaks was accomplished using the ‘addReproduciblePeakSet’ function, with the parameters set as groupBy = ‘celltype’ and pathToMacs2 = pathToMacs2. Further analysis involved utilisation of the ‘getMarkerFeatures’ and ‘getMarkers’ functions to acquire and define marker peaks specific to each cell type.

#### Motif enrichment and TF footprinting identification

The ‘addMotifAnnotations’ function from the ArchR package^17^ was utilised with default parameters for motif annotation, specifying the motifSet as ‘cisbp’ and assigning the name ‘Motif’. Subsequently, motif enrichment analysis was performed using the ‘peakAnnoEnrichment’ function, whereby the top 5 group–specific motifs (including cell type, age, and brain areas) were visualised in a heatmap.

We utilized the ‘getPositions’ function with default parameters to extract the positions of the relevant motifs. Subsequently, we employed the ‘addGroupCoverages’ function and ‘getFootprints’ function to obtain group specific TF footprints. Finally, the TF footprints were visualized using the ‘plotFootprints’ function.

## Data Records

All raw data generated through sciATAC-seq across four age stages of mice in four brain regions has been meticulously documented and deposited in the CNGB Nucleotide Sequence Archive (CNSA)^19^. The specific accession number for this dataset is CNP0004658^20^. The raw data has also been submitted to the NCBI Sequence Read Archive (SRP500857^21^). Furthermore, the barcode information, metadata, cell-GeneScore matrix and cell-type peak matrix had been also uploaded to Figshare (10.6084/m9.figshare.24998411.v3)^22^ as well.

## Technical Validation

We considered four distinct developmental stages – early postnatal (7 P), adult (3 M), middle-aged (12 M), and elderly (18 M) – and four brain regions – OB, CB, PFC, and HP. We conducted sciATAC-seq as illustrated in Fig. 1a. To ensure the robustness of the data, we used 2–8 biological replicates at each developmental stage to construct the sciATAC-seq libraries. The raw sequencing data underwent standard processing, as depicted in Fig. 1b.

**Fig. 2:**
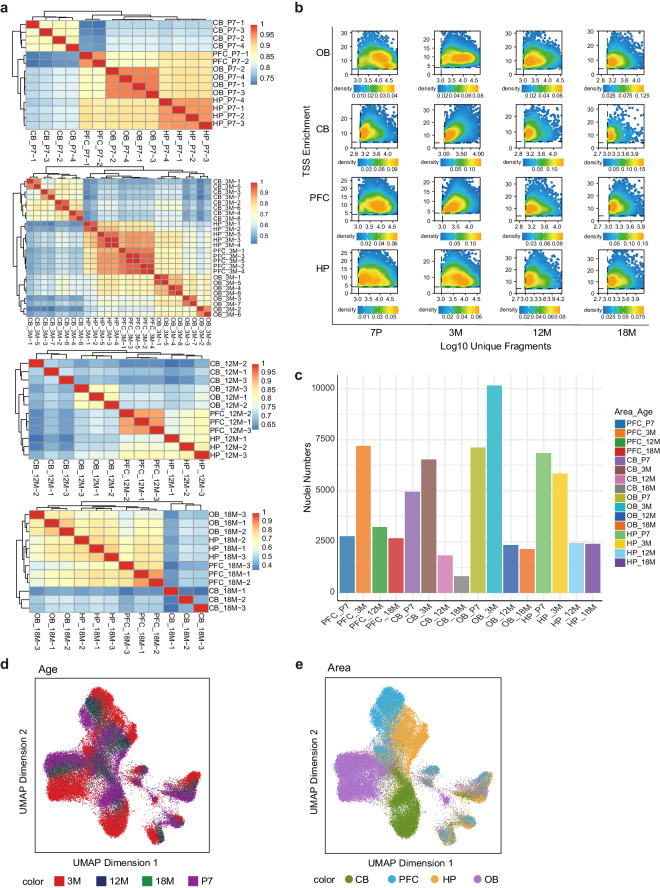
Quality metrics for single-nucleus assay for transposase-accessible chromatin with sequencing (sciATAC-seq). (**a**) Heatmap showing the similarity among the 64 libraries. Correlation analysis was performed among the libraries from the same age, revealing larger correlations among libraries from the same brain region. The heatmap for the four developmental stages, from top to bottom, represents postnatal day 7 (7 P), 3 months (3 M), 12 months (12 M), and 18 months (18 M). (**b**) Quality control (QC) filtering graph depicting the transcription start site (TSS) enrichment scores and unique cell fragments for each cell across different developmental stages and brain regions. (**c**) Filtered nuclear counts for 16 combinations of age and brain regions. (**d**) Uniform manifold approximation and projection (UMAP) displaying the distribution across different developmental stages; distinct colours represent different developmental stages. (**e**) UMAP illustrating the distribution across different brain regions; distinct colours represent different brain regions.

Initially, we applied Pearson correlation coefficient analysis to the 64 libraries to evaluate the similarity among the libraries at different developmental stages. The clustering plot revealed that replicates originating from the same brain region exhibited the highest correlation, affirming the high reproducibility of both biological and technical replicates (Fig. 2a).

Subsequently, we filtered out low-quality cells based on the criteria of minimum unique fragments (nFrags) count ≥ 1000 and transcription start site (TSS) score ≥ 4. Additionally, we applied doublet filtering to the libraries using a filterRatio of 2. We computed the TSS enrichment scores and the number of unique fragments for each cell nucleus in each brain region and at each developmental stage (Fig. 2b, Table 1). The TSS enrichment scores predominantly fell within the range of 8–13, while the number of unique fragments was 1600–9000 (Table 1). Ultimately, a total of 68,721 high-quality cell nuclei were retained across all age groups and brain regions (Fig. 2c, Table 1), identifying a total of 552,385 non-redundant peaks.

**Table 1:** An overview of quality control parameters for the single-nucleus assay for transposase-accessible chromatin with sequencing profiles established for mouse development and ageing.

Time	Area	Library repetition number	Final number of cells	Median fragment	Transcription start site enrichment
7 P	OB	4	7,045	5,967	10.17
7 P	CB	4	4,906	2,231	10.98
7 P	HP	4	6,772	4,831	9.92
7 P	PFC	2	2,740	8,667	11.00
3 M	OB	8	10,088	3,257	8.41
3 M	CB	8	6,500	1,920	12.85
3 M	HP	5	5,800	4,536	9.47
3 M	PFC	5	7,142	5,448	9.55
12 M	OB	3	2,327	2,079	11.08
12 M	CB	3	1,824	1,967	10.28
12 M	HP	3	2,415	2,195	12.57
12 M	PFC	3	3,191	3,240	11.27
18 M	OB	3	2,123	1,770	12.12
18 M	CB	3	804	1,611	10.66
18 M	HP	3	2,393	2,158	12.34
18 M	PFC	3	2,651	2,182	10.09

7 P, postnatal day 7; 3 M, 3 months; 12 M, 12 months; 18 M, 18 months; CB, cerebellum; HP, hippocampus; PFC, prefrontal cortex; OB, olfactory bulb.

Next, we performed clustering and annotation on the quality-controlled 68,721 cell nuclei. We employed optimized iterative LSI to project the cell nuclei into a low-dimensional embedding. Uniform manifold approximation and projection (UMAP) visualisation indicated that gene activity heterogeneity among the cells from different developmental stages was not prominent (Fig. 2d). However, heterogeneity in gene activity among different brain regions was more apparent (Fig. 2e), suggesting distinct genetic activity states among cells in different brain regions, corresponding to varied functional roles in the brain.

**Fig. 3:**
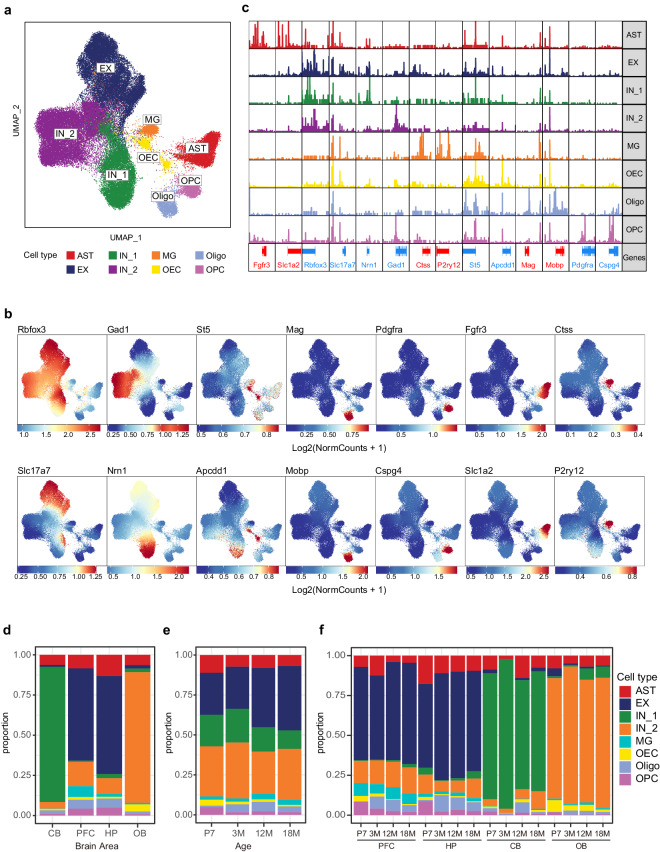
Clustering and annotation of chromatin accessibility and gene expression patterns in the developing and ageing mouse brain. (**a**) Uniform manifold approximation and projection (UMAP) representation of 10 clusters identified by single-cell assay for transposase-accessible chromatin with sequencing (scATAC-seq). Cell colours indicate annotations. (**b**) UMAP visualisation of cell type–specific gene activity scores. (**c**) Genome browser view depicting aggregated scATAC-seq chromatin accessibility profiles of cell type–specific gene loci. (**d**) Histogram displaying cell type proportions across the different brain areas, with colours representing the cell types. (**e**) Histogram displaying cell type proportions across the ages, with colours representing the cell types. (**f**) Histogram showing the distribution of cell proportions across the four brain areas and four ages, totalling 16 combinations, with colours indicating the cell types.

Furthermore, we performed cell annotation using marker gene activity scoring. This analytical approach allowed use to identify 8 distinct cell types (Fig. 3a,b), including AST: (*Fgfr3*^23^ and *Slc1a2*^24^), MG (*P2ry12*^25^ and *Ctss*^26^), Oligo (*Mag*^27^ and *Mobp*^27^), OEC (*Apcdd1*^28^ and *St5*^28^), OPC (*Pdgfra*^27^ and *Cspg4*^27^), EX (*Rbfox3*^29^ and *Slc17a7*^27^), and IN_1(*Rbfox3*^29^ *and Nrn1*^30^) and IN_2 (*Rbfox3*^29^ *and Gad1*^27^). *Rbfox3* serves as a marker for mature neurons, distinguishing neuronal cells from glial cells.

We identified differential accessibility regions (DARs) specific to each of the 10 cell types (Fig. 3c). For instance, signal peaks detected within the TSS region of marker genes *Fgfr3* and *Slc1a2* exhibited specific enrichment in AST. Similarly, signals peaks around *Pdgfra* and *Mobp* were specifically enriched in OPC but not observed in other cell types. These observations strongly indicate the high quality of our data and its alignment with the molecular characteristics of the cells.

We delineated the composition of various cell types across different developmental stages and brain regions. There were notable differences in the distribution of cell types across different brain regions (Fig. 3d), while differences across developmental stages were relatively minor (Fig. 3e). Brain region heterogeneity in cell composition was more pronounced than developmental stage heterogeneity. For instance, the distribution of neuronal subgroups exhibited a high degree of brain region specificity, potentially corresponding to the structural composition and functions of different brain regions. In addition, glial cell proportions were significantly lower than neuronal proportions, and their spatiotemporal distribution characteristics were consistent with existing research findings. For instance, populations of oligodendrocyte precursor cells (OPCs), which are progenitor cells, were most abundant in the brain at the earliest developmental stage, 7 days postnatal (Fig. 3e), consistently across four brain regions (Fig. 3f). However, as time progressed, they gradually decreased during development and aging, =consistent with the consensus that stem cells differentiate into various cell types and their numbers decrease over developmental stages.

**Fig. 4:**
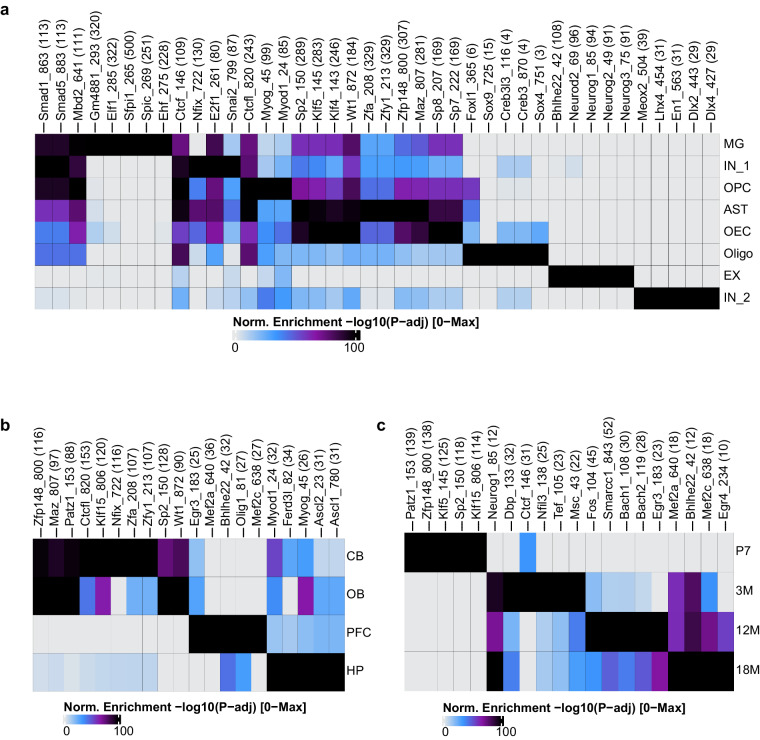
Motif enrichment in marker peaks. (**a**) The heatmap illustrates the cell type–specific enrichment of motifs. (**b**) The heatmap illustrates the brain areas-specific enrichment of motifs. (**c**) The heatmap illustrates the ages–specific enrichment of motifs.

**Fig. 5:**
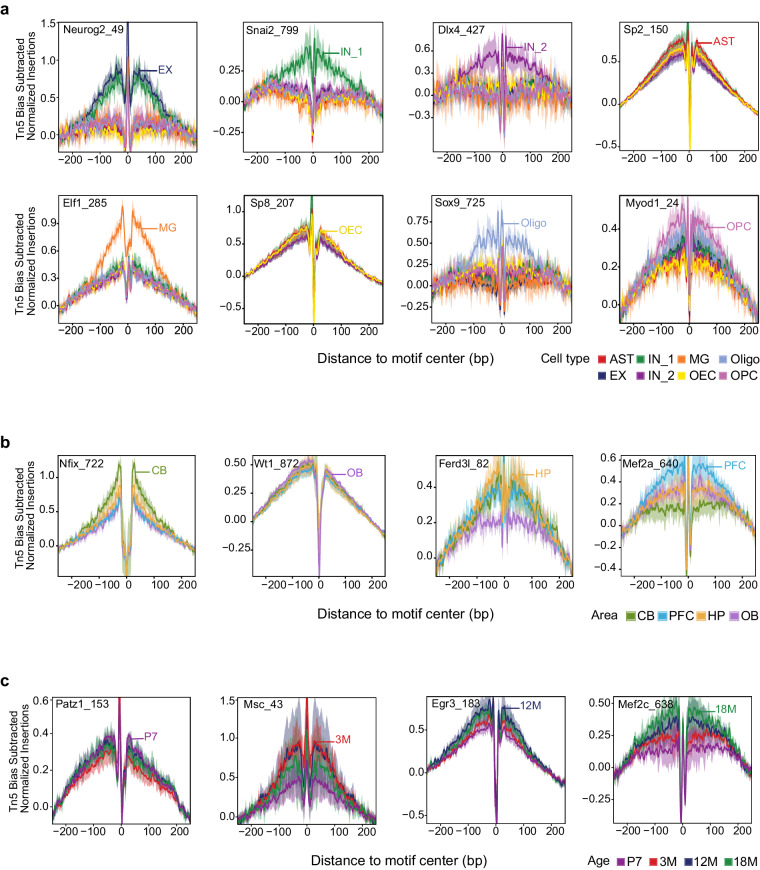
Footprint analysis identifies representative TFs activities in sciATAC-seq data. (**a**) Cell type-specific TFs footprint profiles. (**b**) Brain areas-specific TFs footprint profiles. (**c**) Ages-specific TFs footprint profiles.

We performed peak calling to identify and characterize transcription factor binding sites within chromatin open regions under different conditions (cell types, brain areas, and age stages). Footprint analysis was employed to demonstrate the active transcription factor (TF) binding activity corresponding to these TF binding sites. For instance, in terms of cell types (Figs. 4a, 5a), EX showed enrichment of neurodevelopmental and neurofunctional TFs such as Neurod2, Neurog1, Neurog2, and Neurog3. MG exhibited enriched motifs for *Elf1* and *Sfpi1*^30^, known for their involvement in lymphocyte proliferation and differentiation. Oligo-specific regions demonstrated enriched binding motifs for Sox9, a pivotal TF facilitating progenitor cell differentiation into mature oligodendrocytes^31–33^. In terms of brain areas (Figs. 4b, 5b), binding motifs of the regulator Nfix^34^, associated with early cerebellar progenitor cell differentiation and adult cerebellar neuron function, were specifically enriched in cells derived from the cerebellum. Conversely, binding motifs of the cell differentiation regulator Wt1^35^, involved in olfactory bulb development, were specifically enriched in cells from the olfactory bulb. In terms of ages (Figs. 4c, 5c), binding motifs of *Patz1*^36^, a transcriptional regulator involved in neural stem cell proliferation and maintenance, as well as regulation of early embryonic brain development and neurogenesis, were specifically enriched at the juvenile stage (P7).

In summary, our data robustly recapitulated the enrichment patterns of specific condition-specific TFs reported in previous studies, demonstrating the accuracy of our cell type identification and the high quality of our dataset. Consequently, our dataset serves as a comprehensive reference and valuable resource for further exploration of the epigenetic regulatory mechanisms underlying the development and aging processes across various regions of the mouse brain.

## Usage Notes

The sciATAC-seq data processing pipeline, including read mapping and peak calling, were run on the Linux operating system. All R source code used for downstream data analyses and visualization are provided online (10.6084/m9.figshare.24998411.v3)^22^.

### Supplementary information

Supplementary table

## Data Availability

The codes used to analyze the data in this study were available online (10.6084/m9.figshare.24998411.v3)^22^.
